# Metallic foreign body in the cheek mimicking a chronic facial abscess

**DOI:** 10.11604/pamj.2013.16.136.3522

**Published:** 2013-12-11

**Authors:** Ali Akhaddar, Mohammed Akhiri

**Affiliations:** 1 Department of Neurosurgery, Avicenne Military Hospital, Marrakech, Morocco; 2 University of Mohammed V Souissi, Rabat, Morocco

**Keywords:** Facial abscess, foreign body, cheek, swelling

## Image in medicine

A 4-year-old girl was admitted complaining of recurrent swelling, facial wound discharge and pain in the left cheek for 4 months with difficulty to open her month completely. Her parents report that after a fall, six months later, she presented a small facial laceration that had spontaneously healed. No particular symptom had developed during 2 months. After that time, the facial wound discharge appeared episodically and a facial abscess was suspected and treated with antibiotics by two physicians without improvement. Plain facial radiography revealed a metallic foreign body in the left cheek without osteolysis. No abscess collection was identified by ultrasonography examination. The patient had a mild leukocytosis, while C-reactive protein and sedimentation rates were normal. An approximately 5 × 10 mm metallic fragment that was surrounded by granulation tissue was palpated and extracted surgically and the patient discharged without complications. A retained foreign body in the check after an apparently minor facial injury is an extremely rare event. The present case report reveals the diagnostic and therapeutic challenges and stresses the importance of high degree of suspicion to diagnose retained facial foreign bodies, especially in children, and the need for early surgical exploration, to avoid chronic and potentially life threatening infectious complications.

**Figure 1:**
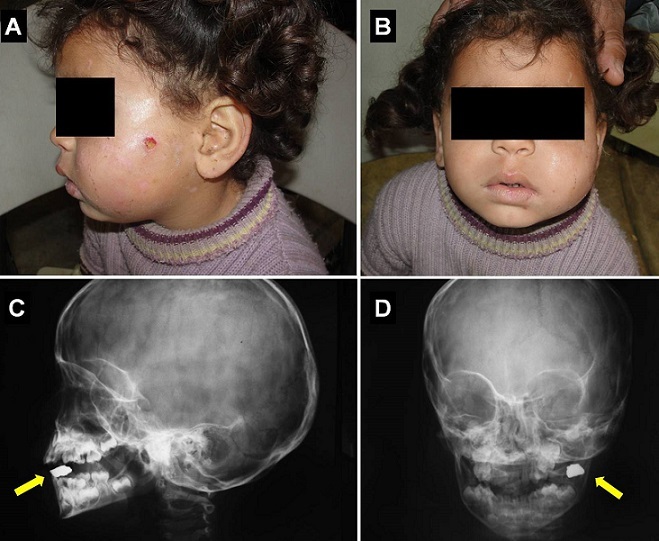
External photograph of the patient presented with left cheek swelling, facial wound discharge and difficulty to open her month completely (A, B); Plain facial radiography revealed a metallic foreign body in the left cheek (arrows) without osteolysis (C, D)

